# From scores to insights: Predicting MT errors using reliable metrics and linguistic typology in slavic languages

**DOI:** 10.1016/j.mex.2025.103613

**Published:** 2025-09-08

**Authors:** Dasa Munkova, Lucia Benkova, Michal Munk, Ľubomír Benko, Petr Hajek

**Affiliations:** a Constantine the Philosopher University in Nitra, Nitra, Slovakia; b University of Pardubice, Pardubice, Czech Republic

**Keywords:** Machine translation, Machine translation evaluation, Machine translation error prediction, Error analysis, Machine Translation error types

## Abstract

Machine Translation (MT) evaluation plays a crucial role in advancing systems translating into morphologically rich, low-resource languages such as Slovak. Existing automatic evaluation methods typically offer a single quality score, lacking insight into specific error types. A novel linguistically informed methodology that predicts the probability of MT error categories by integrating manual annotation with automatic evaluation metrics is proposed. The method builds on a modified MQM framework adapted for Slovak and employs a dataset of English-to-Slovak translations, combining outputs from statistical and neural MT systems with human reference translations. Manual annotations identified five linguistically motivated error categories. Reliability of 68 automatic metrics was assessed using Cronbach’s alpha, correlation coefficients, coefficient of determination (R²), and entropy. Bootstrapped logistic regression models were then developed to predict error occurrence probabilities. The proposed methodology improves the explainability and reliability of automatic MT evaluation by bridging the gap between holistic scoring and detailed error categorization. It significantly reduces the human effort required for quality assessment while maintaining a high degree of linguistic relevance, particularly for complex target languages like Slovak.

• Predicts probabilities of specific MT error categories
• Integrates linguistic expertise with statistical reliability analysis
• Reduces human effort in MT evaluation while preserving linguistic precision

## Graphical abstract

**Figure:**
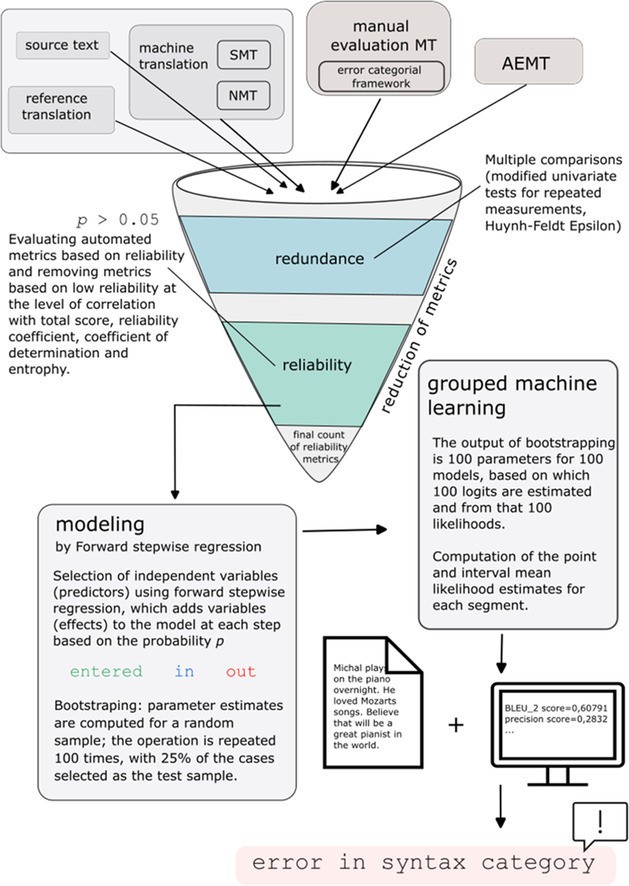

## Specifications table

**Table:** 

**Subject area**	Computer Science
**More specific subject area**	Machine Translation evaluation
**Name of your method**	Prediction of machine translation errors
**Name and reference of original method**	None
**Resource availability**	Python 3 Python libraries for machine translation evaluation metrics (e.g., NLTK or PyTorch) Segment alignment (e.g., HunAlign, LF Aligner or Python libraries) Statistical software (e.g., R, STATISTICA Data Miner or IBM SPSS Modeler)

## Background

Machine translation (MT) evaluation plays a crucial role in improving MT systems, especially when dealing with morphologically rich, low-resource, and highly inflectional languages such as Slovak [1,2]. Existing automatic MT evaluation methods primarily focus on measuring the similarity or edit distance between MT outputs and reference translations (human translations) at lexical, syntactical, semantical, and discourse levels. However, such methods provide an overall score for a MT system, without offering detailed insights into the specific types of errors produced by MT system [3]. In contrast, error analysis serves as a vital process for diagnosing and refining MT models to improve the translation quality and comprehension [4].

To obtain detailed insights into MT model performance, Popovic and Ney [5], as a first, proposed an automatic error analysis procedure based on standard word error rates (WER and PER) combined with linguistic information such as base forms and POS tags. Using these linguistic features, they examined five error categories: inflectional error, reordering errors, missing words, extra words and incorrect lexical choice – following the error typology proposed by Vilar et al [6]. Popović [7] later concluded that most MT error types can by categorized as lexical errors, morphological errors, syntactic errors, semantic errors, orthographic errors, omission errors, addition errors, reordering errors and segments with multiply complex errors. A combination of automated metrics and human judgment, especially for critical semantic errors common in neural MT output, offers the most comprehensive approach MT evaluation [4].

Traditional evaluation methodologies, including human assessments based on error typology like the Vilar et al [6] error taxonomy, have proven insufficient for capturing the complex errors that arise in machine translation into Slavic languages. Slovak exhibits extensive grammatical inflection, flexible word order, and cumulative morphological features such as number, case, gender, and tense [1]. These characteristics complicate automatic error identification and classification and demand a linguistically oriented framework that more accurately reflects translation quality.

The Multidimensional Quality Metrics (MQM) framework [8] offers a more comprehensive and systematic approach to assessing MT system performance. It has been widely adopted in recent in WMT metrics shared tasks and quality estimation tasks as a high-quality human evaluation method [[9], [10], [11]]. However, for Slavic languages, slight modifications and adaptations are necessary as suggested by Klubička et al [12] and Vaňko [13], who emphasized the need to account for fluency (in terms of specific grammatical features common in Slavic languages) and terminology (particularly register).

The methodology proposed in this article integrates methodologies for human assessment of translation quality and automatic metrics for translation evaluation to provide a comprehensive framework for MT quality evaluation.

Despite ongoing advancements, recent research continues to face challenges in seeking a balance between the reliability and explainability of automatic evaluation metrics [14]. In response, we propose a methodology for human-like automatic MT evaluation that estimates the likelihood of potential MT error and explicitly identifies MT error categories based on automatic evaluation metrics.

## Method details

The proposed methodology employs linguistically motivated error typology that is aligned with the core structure of the error typology in the DQF-MQM framework. Within this error typology, the Language dimension (in MQM) corresponds to error categories such as Predication, Syntactic-semantic correlativeness, Compound/complex sentence, and, in part, Modal and communication sentence framework. The Accuracy dimension primarily relates to incorrect meaning in the target text, including omission of lexemes, and is captured within the category of Lexical semantics. This also encompasses Terminology, understood as the inadequate transfer of a term from the source to the target language. The last error category, Style, includes mismatches between the style of the source and target texts and is reflected in the Stylistic compatibility under Lexical semantics [13]. More specifically [1]:

• **Predication** focuses on predicative structure (tense and mode) and agreement categories (person, number and gender).
• **Modal and communication sentence framework** addresses expression of the author's attitude towards the action through modality (negation, necessity, possibility, intention, or obligation) and communication functions (interrogativeness, directiveness, or optativeness).
• **Syntactic-semantic correlativeness** targets morphosyntactic features of nouns, pronouns, numerals, and verbs, as well as word order and other morphosyntactic phenomena.
• **Compound/complex sentences** involve the identification of semantic relations between clauses, cohesion between sentences, and shifts in temporal reference.
• **Lexical semantics** deals with adequate meaning transfer, homonymy, polysemy, terminology, and both semantic and stylistic compatibility.

The central aim of the research methodology is to design a predictive model for MT error detection and classification using automatic evaluation metrics. This model analyzes the MT output at both the word and sentence levels and predicts the likelihood of MT errors within one of the five defined error categories: Predication, Modal and communication sentence framework, Syntactic-semantic correlativeness, Compound/complex sentences, and Lexical semantics.

The proposed methodology contributes to a more efficient, human-like automatic MT evaluation, particularly in identifying and classifying MT errors from an analytical language, English, into a synthetic Slavic language such as Slovak. Moreover, this methodology is both original and generalizable, offering potential applicability across various language pair.

The methodology consists of the following steps:

1. **Obtaining unstructured data and pre-processing**
  All formatting, figures, tables, and other visual elements that might interfere with text alignment were removed from the obtained texts. Since formatting can negatively impact machine translation, only plain text was used. Texts, primarily extracted from the British online newspaper The Guardian, were split into segments, where each segment corresponds to a single sentence.
2. **Generating MT outputs via various systems (e.g. SMT and NMT)**
  Translations were generated using different MT systems (in 2015/2016 and 2018/2019). Two different MT outputs were included in the dataset used for this research: statistical MT and neural MT. The statistical MT output was generated using Google Translate (GT) and the European Commission´s MT system (mt@ec). The neural MT output was generated using the same providers, Google Translate services and the European Commission’s tool (eTranslation), both based on neural networks.
3. **Obtaining reference translations from professional translators**
  Human translations (from English into Slovak) were created by two professional translators in 2020, served as reference translations. As the translation environment, we employed the OSTPERE system (Online System for Translation, Post-Editing, Revision, and Evaluation) [15,16].
4. **Aligning text files at the segment level: Creating a parallel corpus**
  The multiple MT outputs and reference translations were aligned at the sentence level using HunAlign tool [17].
5. **Manual MT evaluation**
  Manual evaluation was conducted by three Slovak language experts, who independently annotated the corpus, sentence by sentence, within the OSTPERE system. Their task was to identify and classify MT errors using a hierarchical categorical framework for Slovak [13]. This manual error analysis served as the foundation, and/or ground truth for subsequent model training and testing.
6. **Automatic MT evaluation using open-source evaluation metrics**
  Python libraries such as NLTK, PyTorch, and others were used to implement these metrics. Most of the chosen metrics are language-independent, making them suitable for model development without requiring deep linguistic or algorithmic knowledge.
7. **Identifying and removing redundant automatic evaluation metrics**
  While using many automatic evaluation metrics (at the beginning - 68 metrics) can improve error coverage, it may also introduce redundancy. Prior to modeling, redundant metrics were identified and removed using various methods: Goodman – Kruskal’s gamma for ordinal associations, Collins’ parsing model for syntactic tree structure, Mauchly’s sphericity test for distributional properties, and conventional reliability estimates such as Cronbach’s alpha and entropy.
8. **Evaluating automatic metric reliability and eliminating low-reliability metrics**
  To evaluate metric reliability, conventional correlation measures were used alongside entropy-based assessment.
9. **Selecting predictors using forward stepwise regression**
  To identify independent variables (predictors), forward stepwise regression was applied. Variables (effects) were added to the model based on a predefined *p* value (entry threshold) and removed if exceeding a removal threshold. Both conservative (*p* < 0.05) and less conservative (*p* < 0.2) thresholds were used to ensure a broad set of potential predictors (metrics).
  The Wald test was employed to evaluate statistical significance of parameter estimates. It offers more precise results, is computationally efficient, and provides a sufficient test score for assessment.
10. **Selecting models with the best predictor subset**
  Models based on optimal subsets of predictors were developed. Model quality was assessed using likelihood ratio (LR) test and Pearson’s chi-square test.
  Marginal analysis (with *df* = 1) and Somers’ D were used to iteratively remove statistically insignificant predictors, thereby optimizing the model for each error category.
11. **Model validation via bootstrapping**
  Validation was carried out using bootstrapping, a resampling method that simulates estimator distributions by treating observed segments as the population. In each of 100 replications, 25 % of the data was used for testing. Since the objective was probability prediction, not classification, Somers’ D was used to evaluate model quality. In logistic regression, Somers’ D estimates the ordinal association between observed binary dependent variable values and predicted probabilities.
12. **Aggregating group machine learning results**
  During the bootstrapping, 100 models were generated for each error category. Using the scores of automatic evaluation metrics, the probability of occurrence of each error category was modeled [18]. Based on these models, the logits for each error category *j* in model *i* were estimated. The logit values for each segment of the analyzed texts are calculated as follows:
  $${\hat{\eta }}_{\mathrm{ij}}=\alpha_{ij}+\beta_{ij}\mathrm{metri}c_{1j}+\cdots +\beta_{ij}\mathrm{metri}c_{\mathrm{kj}},\;i=1,\ldots ,100,\;j=1,\ldots ,J,$$
  where α,β are model parameters, metric represents the selected evaluation metric (various metrics were selected for each error category based on the previous steps), and k is the number of metrics used for error category j and J is the total number of error categories.
13. **Modelling the probability of error category occurrence**
  Using the logits ${\hat{\eta }}_{ij}$ for each model, the probability of error category occurrence $\hat{\pi }$ for segment and model was estimated (for all one hundred models and *j* error categories based):
  $${\hat{\pi }}_{\mathrm{ij}}=\frac{e^{{\hat{\eta }}_{\mathrm{ij}}}}{1+e^{{\hat{\eta }}_{\mathrm{ij}}}},\;i=1,\ldots ,100,\;j=1,\ldots ,J,$$

where ${\hat{\eta }}_{\mathrm{ij}}$ are the logit estimates for error category *j* for model *i*. These probabilities were aggregated across models to obtain the mean and confidence interval (mean±1.96*standarderror). Aggregated model quality was evaluated using *t*-test for independent samples.

This approach provides a scalable alternative to manual MT error analysis and classification. While it estimates the likelihood of specific error categories, human expert verification remains essential. However, similar to how post-editing reduces translation time, the proposed model can significantly reduce the evaluator´s workload by filtering out segments with a low probability of error.

## Method validation

### Parallel and annotated corpus

The proposed methodology was validated using a dataset [19] of newswriting texts sourced from the British online newspaper The Guardian. The training dataset comprises 2036 segments (sentences) produced by SMT and NMT systems each. Each segment is associated with multiple automatic evaluation metric scores and corresponding human annotated error category. Manual error analysis classifies MT errors into five linguistically motivated categories [13]:

• Predication,
• Modal and communication sentence framework,
• Syntactic-semantic correlativeness,
• Compound/complex sentences,
• Lexical semantics.

After manual error analysis, a total of 1818 error segments were identified in NMT outputs and 1551 in SMT outputs (Table 1). The lexico-grammatical distribution of the parallel and annotated corpus is presented in Table 2. In this research, the NLTK was primarily used to compute most of the automatic evaluation metrics.

**Table 1:** Descriptive statistics.

	SMT	NMT
**Error segments**	1551	1818
**Correct segments**	485	218
**Predication**	656	453
**Modal and communication framework**	342	34
**Syntactic-semantic correlativeness**	1074	962
**Compound/complex sentences**	1103	447
**Lexical semantics**	1181	1668

**Table 2:** Lexico-grammatical distribution of the training corpus.

Feature type	ST	SMT	NMT	HT
Average length of sentence (words)	19.42	17.17	17.25	17.90
Average length of words (characters)	4.95	5.57	5.66	5.76
Frequency of long sentences (*w* ≥ 10)	1624	1551	1545	1572
Frequency of short sentences (*w* < 10)	412	485	491	464
Frequency of nouns	8816	9359	9412	10,079
Frequency of adjectives	3163	4475	4432	4646
Frequency of verbs	5209	4160	4358	4406
Frequency of determiners	3942	1915	1876	1984
Frequency of adpositions	4640	3716	3865	4115
Frequency of proper nouns	3411	2323	2324	2260
Frequency of coordinating conjunctions	1246	1314	1294	1346
Frequency of subordinating conjunctions	755	1294	1349	1258
Frequency of interjections	15	26	18	20
Frequency of adverbs	1719	1421	1358	1471
Frequency of pronouns	2620	1049	1252	1412
Frequency of auxiliary	2455	1667	1329	1278
Frequency of numerals	1017	1154	1190	1102
Frequency of particules	1348	507	536	694
Frequency of other POS tags	10	444	380	412

Note: ST – Source Text, SMT – Statistical Machine Translation, NMT – Neural Machine Translation, HT – Human Translation.

For example, when evaluating syntactic-semantic correlativeness of MT output, Benkova and Benko [20] showed that only certain metrics are required - specifically BLEU-4, F-measure, ROUGE-L, and NIST (all n-gram-based), as well as CharacTER (an edit distance-based metric). This finding supports the approach adopted in this study: not all automatic evaluation metrics are necessary for reliable evaluation of MT quality or error identification and classification.

### Reliability of automatic metrics

Examined automatic evaluation metrics were evaluated from a reliability perspective using three distinct approaches: Cronbach's alpha, correlation, and entropy. For each metric, we examined how its removal would impact the overall reliability of the automatic MT evaluation. Specifically, we analyzed changes in the mean (*Mean if deleted*) and variance (*Var. if deleted*), the correlation between the individual automatic metric and the total score (*Metrics-totl. correl*), the coefficient of determination between the metric in automatic MT evaluation and the remaining metrics (*Squared multp. R*), and the revised reliability coefficient after deletion (*Alpha if deleted*).

Most automatic metrics demonstrated stability in variance (*Variance_accuracy* = 57.907; *Variance_error* = 2.392), indicating that they measure translation quality in consistent manner. However, a few evaluation metrics showed divergence in variability: for accuracy metrics BERTscore-f (57.797), Comet (57.774), NIST (45.960), and for error rate metrics TER (1.955) and Torch_WER (1.729).

**Fig. 1:**
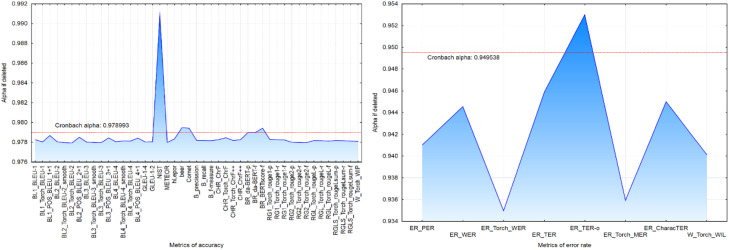
– Cronbach alpha coefficient for accuracy (a) and error rate (b) metrics.

Using the *Alpha if deleted* reliability coefficient*,* four accuracy evaluation metrics - NIST, beer, BERTscore-f, and Comet - were found to reduce overall reliability of automatic MT evaluation (Fig. 1a). Among error rate metrics, TER-o similarly reduced overall reliability score of automatic MT evaluation (Fig. 1b).

**Fig. 2:**
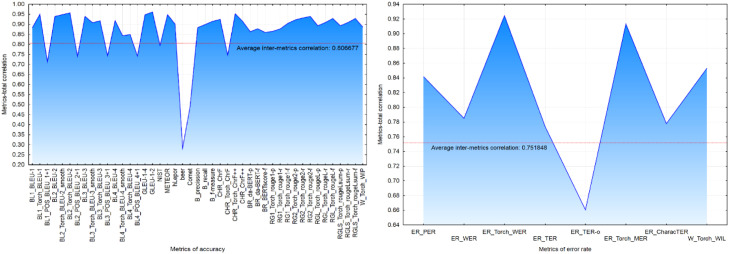
– Coefficient of correlation for accuracy (a) and error rate (b) metrics.

A correlation analysis further revealed low correlations with the total evaluation score (*Metrics-totl. Correl)* for several accuracy metrics: POS_BLEU_1 + 1, POS_BLEU_2 + 1, POS_BLEU_3 + 1, POS_BLEU_4 + 1, Torch_ChrF, beer, Comet (Fig. 2a) and error rate metric TER-o (Fig. 2b). Metrics exhibiting low correlation with the overall score were flagged as suspicious.

**Fig. 3:**
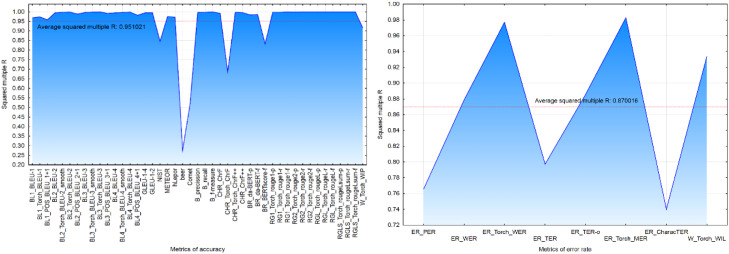
– Coefficient of determination for accuracy (a) and error rate (b) metrics.

The coefficient of determination *Squared multiple R (R2*) measures the degree to which each evaluation metric could be explained by the remaining metrics. Below-average values were observed for accuracy metrics: NIST, beer, Torch_ChrF, Comet, and BERTscore-f (Fig. 3a) and for error rate metrics: TER, CharacTER, and PER metrics (Fig. 3b).

**Fig. 4:**
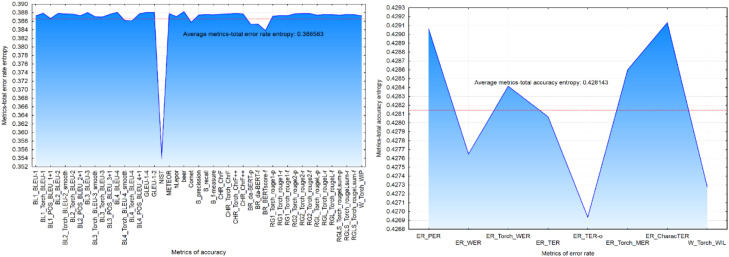
– Entropy for accuracy (a) and error rate (b) metrics.

*Cronbach's alpha* and *correlation* analysis are widely recognized as standard approaches for evaluating the reliability of metrics. However, drawing on the work of Munk et al. [21], we adopted a complementary and less conventional method: entropy analysis. In their study, Munk et al. [21] evaluated both accuracy metrics (such as accuracy, recall, F-measure, and BLEU) and error rate metrics (including WER, PER, and CDER), concluding that entropy provides more reliable insights into metric stability than traditional estimators. In this research, we replicated and extended Munk et al.’s [21] approach by applying entropy to a broader set of automatic MT evaluation metrics (Fig. 4). Entropy was calculated at the segment level, and the average entropy across all segments was computed separately for accuracy and error rate metrics.

The entropy analysis revealed several metrics with high disorder, which were flagged as suspicious (potentially unreliable) and subsequently excluded from the model. These included: NIST, BERTscore-f, Comet, da-BERT-p, da-BERT-f, Torch_BLEU-4_smooth, and Torch_BLEU-4 (accuracy metrics) and TER-o, TER, WER, and Torch_WIL (error rate metrics). These metrics exhibit below-average entropy values (Table 3 and Table 4).

Entropy was treated as one of several diagnostic variables in the overall reliability assessment. To ensure a conservative and robust exclusion strategy, a metric was removed from the final model only if it demonstrated poor performance across all four reliability indicators: low correlation with the total score (*Metrics-totl.correl*), higher reliability coefficient (*Alpha if deleted*), low coefficient of determination (*Squared multp. R*), and low entropy value (*Metrics-totl. err. r. ent.*).

Based on the specified exclusion criteria – considering all four reliability indicators – two accuracy metrics, NIST and Comet, were removed from the model (Table 3). In contrast, none of the error rate metrics fulfilled the exclusion condition across all four indicators and were therefore retained in the model (Table 4).

**Table 3:** – Results of the reliability estimation for selected accuracy metrics (Summary for scale: *Average inter-metrics correlation* = 0.8067, *Average squared multiple R* = 0.9510, *Cronbach alpha* = 0.9790, *Standardized alpha* = 0.9930, *Average metrics-total accuracy entropy* = 0.386563).

	Metrics-totl. correl.	Squared multp. R	Alpha if deleted	Metrics-totl. err. r. *ent*.
NIST	**0.7952**	**0.8460**	**0.9909**	**0.3545**
beer	**0.2830**	**0.2757**	**0.9795**	0.3882
BR_BERTscore-f	0.8599	**0.8319**	**0.9794**	**0.3838**
Comet	**0.4905**	**0.5199**	**0.9794**	**0.3858**
BR_da-BERT-p	0.8630	0.9841	0.9790	**0.3853**
BR_da-BERT-f	0.8782	0.9855	0.9790	**0.3853**
BL1_POS_BLEU_1 + 1	**0.7122**	0.9581	0.9787	0.3866
BL2_POS_BLEU_2 + 1	**0.7394**	0.9888	0.9785	0.3873
CHR_Torch_ChrF	**0.7459**	**0.6849**	0.9785	0.3877
BL3_POS_BLEU_3 + 1	**0.7432**	0.9926	0.9784	0.3876
BL4_POS_BLEU_4 + 1	**0.7411**	0.9820	0.9784	0.3877
BL4_Torch_BLEU-4_smooth	0.8426	0.9970	0.9781	**0.3862**
BL4_Torch_BLEU-4	0.8489	0.9982	0.9781	**0.3861**
W_Torch_WIP	0.8866	**0.9169**	0.9781	0.3873

Note: marked < Average inter-metrics correlation, marked < Average squared multiple R, marked > Cronbach alpha, marked < Average metrics-total accuracy entropy.

**Table 4:** – Results of reliability estimation for selected error rate (Summary for scale: *Average inter-metrics correlation* = 0.7518, *Average squared multiple R* = 0.8700, *Cronbach alpha* = 0.9495, *Standardized alpha* = 0.9500, *Average metrics-total error rate entropy* = 0.428143).

	Metrics-totl. correl.	Squared multp. R	Alpha if deleted	Metrics-total acc. *ent*.
ER_TER-o	**0.660718**	0.885335	**0.953004**	**0.426936**
ER_TER	0.773748	**0.797358**	0.945861	**0.428070**
ER_CharacTER	0.777917	**0.740153**	0.945009	0.429133
ER_WER	0.785081	0.878429	0.944563	**0.427650**
ER_PER	0.841643	**0.765346**	0.941026	0.429064
W_Torch_WIL	0.852998	0.933663	0.940124	**0.427279**

Note: marked < Average inter-metrics correlation, marked < Average squared multiple R, marked > Cronbach alpha, marked < Average metrics-total accuracy entropy.

The integration of bootstrapped logistic regression models with a rigorous, multi-criteria metric selection process ensured the robustness and predictive accuracy of the final models. This combined methodology not only enhances the reliability of automatic MT evaluation but also confirms its practical applicability for modeling error probability. Furthermore, the validated approach demonstrates substantial potential to reduce manual labor in MT evaluation (error analysis and classification) by reliably identifying segments with a high likelihood of translation errors.

## Limitations

Despite the methodological rigor and innovation introduced in this study, several limitations must be acknowledged. The manual annotation process involved only three Slovak language experts. While this ensured consistency, it may limit generalizability due to the absence of broader inter-annotator agreement analysis. The strictness of the four-indicator exclusion criterion, while methodologically sound, could limit metric diversity in future applications. Despite automation in evaluation, the methodology still depends on a foundational corpus of manually annotated translations. The logistic regression models output probabilities rather than categorical classifications. While this supports risk-based filtering, it necessitates continued human validation, especially in critical applications (e.g., legal or medical translation). Although bootstrapping provides statistical robustness, the use of 100 replications and 25 % test samples may not capture all possible variations in larger or more diverse corpora.

## CRediT authorship contribution statement

**Dasa Munkova:** Supervision, Writing – review & editing. **Lucia Benkova:** Conceptualization, Methodology, Writing – original draft. **Michal Munk:** Conceptualization, Methodology, Writing – review & editing. **Ľubomír Benko:** Conceptualization, Methodology, Writing – original draft. **Petr Hajek:** Supervision, Writing – review & editing.

## Declaration of competing interest

The authors declare that they have no known competing financial interests or personal relationships that could have appeared to influence the work reported in this paper.

## Data Availability

The dataset analyzed during the current study is available in the Mendeley Data repository under 10.17632/zx3vt8r26k.1.
